# Neutrophil dysregulation in sepsis-induced acute lung injury: mechanistic framework and therapeutic targeting by natural products

**DOI:** 10.3389/fphar.2026.1925322

**Published:** 2026-10-14

**Authors:** Huilin Luo, Jinyuan Lin, Honglei Shen, Dongqin Huang, Maoyao Ling, Liu Ye

**Affiliations:** 1 Department of Anesthesiology, Guangxi Medical University Cancer Hospital, Nanning, China; 2 Guangxi Engineering Research Center for Tissue & Organ Injury and Repair Medicine, Nanning, China; 3 Guangxi Health Commission Key Laboratory of Basic Science and Prevention of Perioperative Organ Dysfunction, Nanning, China; 4 Guangxi Clinical Research Center for Anesthesiology, Nanning, China

**Keywords:** immune dysregulation, natural products, neutrophil extracellular traps, neutrophil heterogeneity, sepsis-induced acute lung injury

## Abstract

Sepsis-induced acute lung injury (SALI) is an important complication of sepsis associated with substantial morbidity and mortality, yet effective targeted therapies remain limited. Neutrophils are central effectors of innate immunity and play indispensable roles in early antimicrobial defense. However, during sepsis, persistent inflammatory stimulation drives neutrophil dysregulation, leading to excessive pulmonary infiltration, aberrant activation, delayed apoptosis, neutrophil extracellular trap (NET) formation, and immune imbalance, all of which contribute to alveolar-capillary barrier disruption and lung injury progression. Emerging evidence indicates that neutrophil heterogeneity in SALI is shaped by multiple molecular regulators rather than by maturation status alone. Trafficking receptors, activation-associated markers, disease-associated transcriptional states, and intercellular signaling pathways—including CXCR4, CXCR2, CD11b, CD16, CD177, Fth1, Prok2, CD64/CD271, PD-L1, and Notch1-related signaling—collectively regulate neutrophil trafficking, activation, effector responses, and crosstalk with endothelial and immune cells. In this review, we summarize the molecular determinants underlying neutrophil functional heterogeneity and discuss the dual role of neutrophils in host defense and tissue injury in SALI. We also highlight the major mechanisms of neutrophil-mediated lung damage and summarize emerging evidence that natural products can alleviate SALI by modulating neutrophil recruitment, oxidative stress, inflammatory signaling, and NET formation. Targeting neutrophil dysregulation represents a potential therapeutic approach that requires further mechanistic and clinical validation.

## 1 Introduction

Sepsis is a life-threatening syndrome characterized by a dysregulated host response to infection that results in organ dysfunction (van der Poll et al., 2017). Among the affected organs, the lung is particularly vulnerable and closely linked to the onset and progression of sepsis (Xie et al., 2020). During sepsis-induced acute lung injury (SALI), invading pathogens and their products, such as bacteria and endotoxins, trigger the release of inflammatory mediators and activate multiple immune and structural cells, including alveolar macrophages, epithelial cells, lymphocytes, and neutrophils. This process culminates in an uncontrolled inflammatory response, severe alveolar–capillary barrier disruption, and pulmonary dysfunction (Sakr et al., 2015; van Vught et al., 2016; Mohsin et al., 2021; Li et al., 2025).

Neutrophils are central effectors in the pathogenesis of acute lung injury/acute respiratory distress syndrome (ALI/ARDS) and play a particularly important role in SALI (Wang et al., 2022). Although neutrophil activation is a shared feature of different forms of ALI/ARDS, sepsis imposes additional systemic inflammatory and infectious stimuli that drive more profound neutrophil dysregulation. This exaggerated response not only amplifies pulmonary inflammation and tissue damage but also contributes to systemic immune imbalance and multiple organ dysfunction, highlighting the distinct pathological features of SALI compared with other causes of ALI/ARDS (Xu et al., 2025). Recent studies further suggest that neutrophils are highly heterogeneous and can adopt diverse functional phenotypes during disease progression (Janicova and Relja, 2021).

As an important pulmonary complication of sepsis, SALI is associated with substantial morbidity and mortality, while effective targeted pharmacological therapies remain lacking (Vincent et al., 2014; van Vught et al., 2016). Therefore, identifying novel therapeutic strategies is of considerable clinical importance. In recent years, natural products have attracted increasing attention for their potential pharmacological relevance to SALI and their multitarget regulatory properties. Unlike single-target agents, natural compounds may simultaneously modulate multiple pathogenic pathways involved in SALI, including inflammatory signaling, oxidative stress, and neutrophil extracellular trap (NET) formation. For example, 1,3,6-tri-O-galloyl-β-D-glucose (TGG) has been reported to attenuate inflammation and oxidative stress by targeting neutrophil IFIT1, thereby alleviating sepsis-induced lung injury (Zhao et al., 2026). Rosavin also exerts protective effects by suppressing excessive inflammation, reducing NET formation, and inhibiting MAPK/ERK/p38/JNK signaling (Gao et al., 2023).

Accumulating evidence indicates that natural products can modulate neutrophil recruitment, activation, oxidative stress, and NET formation, thereby alleviating lung injury in sepsis. However, a focused review on neutrophil-targeting natural products in SALI is still lacking. Therefore, this review summarizes the role of neutrophils in SALI, with particular emphasis on their functional heterogeneity and pathogenic mechanisms, and further discusses the therapeutic potential and underlying mechanisms of natural products that target neutrophil-related pathways in SALI.

## 2 Literature search strategy

This narrative review was informed by searches of PubMed, Web of Science, and Scopus from database inception to August 2026. Search terms included combinations of “sepsis-induced lung injury,” “sepsis-associated acute respiratory distress syndrome,” “acute lung injury,” “neutrophil,” “neutrophil heterogeneity,” “neutrophil extracellular traps,” “natural products,” “herbal medicine,” and the names of individual compounds or formulations. Reference lists of relevant original articles and reviews were also examined to identify additional eligible studies.

For the natural-product section, eligible studies were required to be peer-reviewed full-length articles that investigated a defined natural compound, extract, or herbal formulation in a sepsis or lung-injury model and reported either a pulmonary outcome or a directly assessed neutrophil-related outcome. Neutrophil-related outcomes included recruitment, infiltration, activation, phagocytosis, apoptosis, ROS production, platelet–neutrophil interactions, and NET formation. Studies reporting both pulmonary outcomes and direct neutrophil or NET measurements were prioritized. Mechanistic studies involving pathway inhibition, genetic manipulation, overexpression, rescue experiments, or cell-based functional assays were also prioritized but were not required for inclusion.

Studies using general LPS-induced ALI models were included only when they provided relevant evidence regarding pulmonary injury and neutrophil-associated mechanisms. Studies limited to nonspecific systemic inflammatory outcomes, without pulmonary or neutrophil-related assessments, were excluded from the principal pharmacological evidence. Reviews and general pathway studies were used only to provide background context and were not treated as direct pharmacological evidence for an individual natural product. Because this was a narrative rather than a systematic review, the search was not intended to be exhaustive and no formal risk-of-bias assessment or quantitative synthesis was performed.

## 3 Phenotypic and functional heterogeneity of neutrophils in SALI

Neutrophil heterogeneity in SALI reflects dynamic differences in maturation, anatomical localization, trafficking, activation, metabolism, survival, and immune regulation. During sepsis, neutrophils undergo continuous phenotypic and functional transitions as they are released from the bone marrow, enter the circulation, become retained within the pulmonary microvasculature, migrate into the lung interstitium, and ultimately accumulate in the alveolar space. These spatial transitions are accompanied by changes in chemokine receptors, adhesion molecules, transcriptional programs, metabolic activity, lifespan, and effector functions. Therefore, individual molecular markers do not necessarily define discrete or stable neutrophil subsets; rather, they may indicate transient, overlapping, and context-dependent cellular states shaped by disease stage and the pulmonary microenvironment. In this section, we summarize neutrophil heterogeneity in SALI according to trafficking- and maturation-associated markers, functional surface phenotypes, disease-associated transcriptional states, and neutrophil–endothelial communication (Figure 1). The principal characteristics and interpretive limitations of these markers are summarized in Table 1.

**FIGURE 1:**
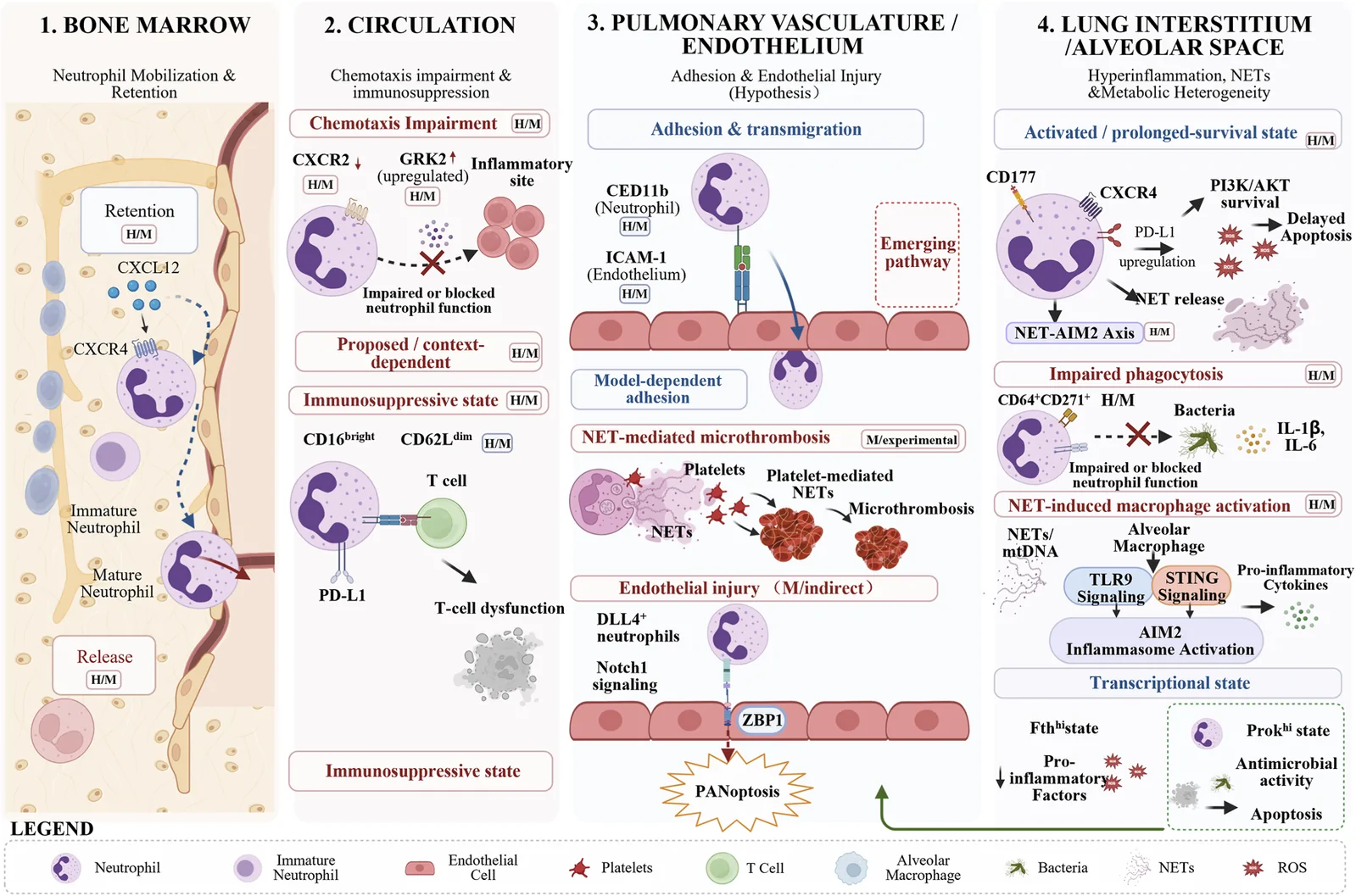
Spatial and functional framework of neutrophil dysregulation in SALI. 1. Neutrophil phenotypes change dynamically across the bone marrow, circulation, pulmonary vasculature/endothelium, and lung interstitium/alveolar space, reflecting context-dependent states rather than discrete stable subsets. 2. In the bone marrow, the CXCL12-CXCR4 axis maintains retention, whereas CXCR2 promotes mobilization. 3. In circulation, reduced CXCR2 expression and GRK2-mediated desensitization may impair chemotaxis, while CD16^bright^CD62L^dim^ and PD-L1^+^ neutrophils may contribute to immunoregulatory dysfunction. 4. In the pulmonary vasculature, CD11b-ICAM-1-mediated adhesion/transmigration, NETs, platelet-neutrophil interactions, and DLL4-Notch1/ZBP1-linked PANoptosis are depicted as canonical, experimental, or emerging mechanisms. 5. In the lung interstitium/alveolar space, activated/prolonged-survival neutrophils exhibit increased CXCR4 expression, PD-L1-PI3K/AKT survival signaling, delayed apoptosis, enhanced NET release, impaired phagocytosis, and NET-driven macrophage activation via TLR9-STING-AIM2; single-cell analyses further identify Fth1^hi^ and Prok2^hi^ transcriptional states. 6. Blue, red, purple, green, and black denote protective/homeostatic, pathogenic/experimental, activation/phenotype, transcriptional, and other interaction/outcome categories, respectively. 7. Solid black, blue dotted, and red dotted arrows indicate canonical/validated, experimental model-dependent, and proposed/indirect/context-dependent mechanisms. 8. H, human evidence; M, mouse/animal evidence; H/M, evidence supported by both human and mouse/animal studies; experimental, experimental animal evidence; proposed, hypothesis-driven mechanism; model-dependent, model-restricted finding; indirect, indirect evidence. 9. Key points: CXCR4 mediates retention via the SDF-1/CXCL12 axis, CXCR2 promotes mobilization, and these trafficking programs should be interpreted as dynamic states rather than stable neutrophil subsets. 10. Abbreviations: NETs, neutrophil extracellular traps; ROS, reactive oxygen species; mtDNA, mitochondrial DNA; SALI, sepsis-induced acute lung injury; scRNA-seq, single-cell RNA sequencing. 11. Created in BioRender: https://BioRender.com/qyxwqg8.

**TABLE 1:** Summary of neutrophil markers and associated phenotypes in SALI.

Marker/phenotype	Category	Principal role	Relevance to SALI or lung injury	Interpretation/limitations	References
CXCR4	Trafficking and maturation receptor	Bone-marrow retention, aging, homing, and clearance	CXCR4^hi^ states are associated with pulmonary retention, glycolysis, ROS production, and NET formation	Dynamic and context-dependent; not a stable subset marker	Liu D. et al. (2023)
CXCR2	Trafficking receptor	Chemokine-directed neutrophil recruitment	Required for LPS-induced pulmonary neutrophil recruitment and vascular permeability	CXCR2 was also expressed on pulmonary endothelial and epithelial cells; the effect was not neutrophil-specific	Reutershan et al. (2006)
CD11b	Adhesion and activation marker	Adhesion, crawling, transmigration, phagocytosis, and activation	Promotes pulmonary sequestration and transendothelial migration	Broad, rapidly inducible marker; not subset-specific	Gao et al. (2005), Park et al. (2019)
CD16/CD62L	Maturation and functional phenotype	Distinguishes immature, mature, and immunoregulatory states	CD16^bright/^CD62L^dim^ neutrophils show immunoregulatory features in sepsis-associated ARDS	Observational clinical evidence; functional causality remains unconfirmed	Zhang et al. (2024)
CD177	Migration and activation phenotype	PECAM-1-dependent transmigration and proteinase-3 binding	Associated with oxidative stress and NET-related injury in experimental sepsis	Interindividual variability; evidence is predominantly experimental	Wang et al. (2025)
Fth1^high^	Transcriptional state	Iron homeostasis, oxidative stress, inflammation, and prolonged survival	Enriched in inflamed lung tissue in experimental ALI	Primarily defined in LPS-induced ALI mice, with limited validation in a small BALF cohort	Wang et al. (2022)
Prok2^high^	Transcriptional state	Antimicrobial and metabolic programs	Predominantly localized to the pulmonary vascular compartment in experimental ALI	Primarily defined in LPS-induced ALI mice, with limited validation in a small BALF cohort	Wang et al. (2022)
CD64^+^/CD271^+^	Disease-associated functional phenotype	Impaired phagocytosis and enhanced inflammatory signaling	Associated with defective bacterial clearance and pulmonary inflammation in experimental sepsis	Emerging phenotype; lineage, dynamics, and clinical relevance remain unclear	Fu et al. (2024)
PD-L1^+^	Immunoregulatory and survival phenotype	Delayed neutrophil apoptosis and prolonged inflammatory activity	Promotes neutrophil persistence and experimental sepsis-induced lung injury	Experimental evidence; clinical relevance remains uncertain	Wang J. F. et al. (2021)
Neutrophil DLL4/endothelial Notch1	Intercellular signaling pathway	Neutrophil-endothelial inflammatory communication	Associated with endothelial dysfunction, barrier injury, and proposed PANoptosis	Notch1 is endothelial rather than neutrophil-specific; mechanism remains experimental	Jin et al. (2025)

Abbreviations: ALI, acute lung injury; NET, neutrophil extracellular trap; ROS, reactive oxygen species; SALI, sepsis-induced acute lung injury.

### 3.1 Trafficking, maturation, adhesion, and activation-associated markers

#### 3.1.1 CXCR4

CXCR4 is a chemokine receptor that regulates neutrophil retention, homing, aging, and clearance through its interaction with CXC12. It is highly expressed in immature neutrophils retained within the bone marrow and is re-expressed by aged circulating neutrophils, facilitating their return to the bone marrow for clearance. During neutrophil maturation, reduced CXCR4 signaling, together with increased CXCR2 activity, promotes neutrophil mobilization from the bone marrow into the circulation (De Filippo and Rankin, 2018). CXCR4 expression therefore reflects both maturation and trafficking status rather than a single, stable neutrophil identity.

During sepsis and inflammatory lung injury, increased CXCR4 expression may promote neutrophil retention within the pulmonary microvasculature and subsequent accumulation in injured lung tissue (Lerman and Kim, 2015). CXCR4^hi^ neutrophils have also been associated with enhanced PFKFB3-dependent glycolysis, ROS production, and NET formation. These processes may contribute to endothelial injury, microvascular thrombosis, and impaired pulmonary perfusion (Liu D. et al., 2023). Pharmacological inhibition of CXCR4 attenuated inflammatory signaling, NET-associated injury, and pulmonary pathological changes in experimental models (Lou et al., 2022).

Nevertheless, CXCR4^hi^ neutrophils should not automatically be regarded as an independently defined pathogenic subset. CXCR4 expression varies according to neutrophil maturation, aging, anatomical localization, and inflammatory exposure. Its functional significance should therefore be interpreted in combination with additional phenotypic, transcriptional, spatial, and functional evidence.

#### 3.1.2 CXCR2

CXCR2 is predominantly expressed by mature neutrophils and mediates their mobilization and recruitment in response to ELR^+^ CXC chemokines, including CXCL1, CXCL2, CXCL5, and CXCL8 (De Filippo and Rankin, 2018). The reciprocal regulation of CXCR2 and CXCR4 contributes to the balance between neutrophil retention in the bone marrow and release into the peripheral circulation. After neutrophils enter the bloodstream, CXCR2 signaling directs their migration toward inflammatory sites.

In SALI, excessive production of CXCR2 ligands promotes neutrophil recruitment to the lung, pulmonary microvascular sequestration, and migration across the endothelial and epithelial barriers (Reutershan et al., 2006). Accumulated neutrophils subsequently release ROS, proteases, inflammatory mediators, and NETs, thereby aggravating alveolar–capillary barrier disruption and pulmonary edema (Zhang et al., 2023). Accordingly, inhibition of the CXCR2 axis may reduce excessive neutrophil infiltration and tissue injury (Zhang et al., 2023).

However, the function of CXCR2 in sepsis is stage- and context-dependent. Sustained systemic inflammation may induce G protein-coupled receptor kinase 2 (GRK2)-dependent CXCR2 desensitization, impairing directed neutrophil migration toward infectious foci and reducing bacterial clearance (Ishii et al., 2012; Martin-Fernandez et al., 2021). Therefore, CXCR2-mediated migration can be either protective or pathogenic depending on the anatomical compartment, disease stage, and balance between antimicrobial defense and tissue injury (Su et al., 2021). CXCR2 expression should consequently be interpreted as a dynamic trafficking-associated feature rather than a marker of a fixed neutrophil subset.

#### 3.1.3 CD11b

CD11b forms the Mac-1 integrin complex with CD18 and plays an essential role in neutrophil adhesion, crawling, transendothelial migration, phagocytosis, and cellular activation (Janicova and Relja, 2021). CD11b is stored intracellularly in resting neutrophils and is rapidly mobilized to the cell surface following activation. Increased surface CD11b expression is therefore commonly used as an indicator of neutrophil activation and enhanced adhesive capacity.

During sepsis, increased CD11b expression facilitates neutrophil adhesion to pulmonary endothelial cells and sequestration within the narrow pulmonary capillary network. This process promotes subsequent migration into the interstitial and alveolar compartments, where activated neutrophils release proteases, ROS, cytokines, and NETs (Yang et al., 2021). Excessive CD11b-dependent adhesion and activation may therefore aggravate endothelial dysfunction, alveolar–capillary barrier injury, and pulmonary inflammation (Zhang et al., 2023). Experimental inhibition of CD11b reduced pulmonary neutrophil infiltration and attenuated lung injury (Gao et al., 2005; Park et al., 2019).

Nevertheless, CD11b is a broadly expressed activation and adhesion molecule rather than a subset-specific marker. Its expression varies rapidly in response to inflammatory stimulation and should be interpreted together with neutrophil localization, maturation status, and functional measurements.

### 3.2 Functional state-associated surface phenotypes

#### 3.2.1 CD16

CD16, also known as Fcγ receptor III, contributes to immune-complex recognition and neutrophil effector functions. Its expression changes during neutrophil maturation and activation. CD16^dim^ neutrophils generally include immature cells, whereas mature circulating neutrophils commonly exhibit a CD16^bright^ phenotype. Based on the combined expression of CD16 and CD62L, mature neutrophils may be further separated into CD16^bright^CD62L^bright^ and CD16^bright^CD62L^dim^ populations, which have been associated with distinct inflammatory and immunoregulatory functions (Williams and Chambers, 2014; Zhang et al., 2024).

CD16^bright^CD62L^dim^ neutrophils have been reported to suppress T-cell proliferation and cytokine production during severe systemic inflammation, suggesting a possible contribution to sepsis-associated immune dysfunction (Pillay et al., 2012). In contrast, other CD16-defined populations may retain inflammatory, migratory, or antimicrobial functions. These phenotypes may vary across disease stages and can be affected by emergency granulopoiesis, neutrophil aging, activation, and proteolytic shedding of surface molecules.

Consequently, CD16 expression alone is insufficient to establish a stable or functionally uniform neutrophil subset. CD16 should be evaluated together with CD62L, maturation markers, transcriptional profiles, and direct functional assessments, particularly when interpreting its significance in SALI.

#### 3.2.2 CD177

CD177 is a glycosylphosphatidylinositol-anchored surface protein expressed by a proportion of mature neutrophils. It participates in neutrophil adhesion and transendothelial migration through interactions with endothelial platelet endothelial cell adhesion molecule-1 and also serves as a surface-binding partner for proteinase 3 (Bai et al., 2017). The proportion of CD177-expressing neutrophils varies considerably among individuals and may increase during infection and systemic inflammation.

In experimental sepsis-related lung injury, increased CD177 expression has been associated with neutrophil activation, oxidative stress, and NET formation. Blocking CD177 reduced NET-associated inflammation, neutrophil-mediated tissue injury, and pulmonary pathological changes in LPS- and CLP-induced models (Wang et al., 2025). These observations suggest that CD177 may identify an activated neutrophil state with enhanced migratory and tissue-damaging capacity under specific inflammatory conditions.

However, CD177 expression alone does not define a stable pathogenic lineage. Its expression is influenced by genetic factors, maturation, inflammatory stimulation, and disease context. Moreover, evidence supporting its role in SALI is currently derived predominantly from experimental models, and its functional relevance in patients with sepsis-associated ARDS requires further validation.

### 3.3 Disease-associated transcriptional and functional phenotypes

#### 3.3.1 Fth1^hi^ and Prok2^hi^ neutrophils

Single-cell RNA sequencing has revealed multiple neutrophil transcriptional states that cannot be adequately distinguished using conventional surface markers. Among these, Fth1^hi^ and Prok2^hi^ neutrophils have been identified in experimental acute lung injury and display different spatial distributions and functional programs (Wang et al., 2022).

Fth1^hi^ neutrophils are enriched in inflamed lung tissue and exhibit transcriptional features associated with iron homeostasis, oxidative stress, inflammation, and resistance to apoptosis. Ferritin heavy chain 1, encoded by Fth1, regulates intracellular iron storage and may influence ROS generation and inflammatory cell injury. The prolonged survival and inflammatory activity of Fth1^hi^ neutrophils may facilitate their persistence in injured lung tissue and amplify local inflammation (Wang et al., 2022).

In contrast, Prok2^hi^ neutrophils are primarily located within the pulmonary vascular compartment and show transcriptional programs related to antimicrobial defense and metabolic activity (Wang et al., 2022). These findings indicate that neutrophils occupying different pulmonary compartments may acquire distinct functional states during lung injury.

However, Fth1^hi^ and Prok2^hi^ populations were defined mainly on the basis of transcriptional patterns in experimental models. They should therefore be regarded as context-dependent cellular states rather than fixed neutrophil subsets. Their developmental relationships, stability over time, and functional relevance in human SALI remain to be established through spatial, protein-level, lineage-tracing, and functional studies.

#### 3.3.2 CD64^+^CD271^+^ neutrophils

CD64, or Fcγ receptor I, is expressed at low levels on resting neutrophils but is rapidly upregulated during bacterial infection and systemic inflammation. Neutrophil CD64 is widely used as a biomarker of bacterial infection and sepsis (Patnaik et al., 2020). However, CD64 expression primarily reflects activation and inflammatory exposure and does not independently define a distinct neutrophil subset.

Co-expression of CD64 and CD271 has been reported to identify a disease-associated neutrophil phenotype characterized by impaired phagocytosis, enhanced inflammatory signaling, and increased cytokine production (Fu et al., 2024). These cells may contribute simultaneously to insufficient pathogen clearance and excessive pulmonary inflammation. Experimental inhibition of CD271 improved bacterial clearance, reduced inflammatory responses, and attenuated lung injury, suggesting that CD271-related signaling may participate in neutrophil dysfunction during sepsis.

These findings support the presence of a dysfunctional CD64^+^CD271^+^ neutrophil state but do not establish a distinct developmental lineage. Additional studies are needed to determine its origin, temporal dynamics, anatomical distribution, and clinical significance in patients with SALI or sepsis-associated ARDS.

#### 3.3.3 PD-L1^+^ neutrophils

Programmed death-ligand 1 (PD-L1) can be induced on neutrophils by inflammatory mediators present during sepsis. PD-L1 expression identifies an immunoregulatory state in which neutrophils may influence both innate inflammation and adaptive immune suppression. Through engagement with programmed cell death protein 1 (PD-1) on lymphocytes, PD-L1^+^ neutrophils can inhibit T-cell activation, proliferation, and cytokine production, potentially contributing to sepsis-associated immunosuppression (de Kleijn et al., 2013; Liu et al., 2024).

PD-L1 signaling may also affect neutrophil survival. Available evidence suggests that PD-L1 expression can delay neutrophil apoptosis through PI3K/AKT-related signaling, thereby prolonging neutrophil persistence and inflammatory activity (Wang J. F. et al., 2021; Xiao et al., 2025). Thus, PD-L1^+^ neutrophils may contribute to both persistent pulmonary inflammation and systemic immune dysfunction.

Nevertheless, PD-L1 expression is dynamic and may differ according to disease severity, inflammatory exposure, and the immunological phase of sepsis. PD-L1 should therefore be considered a functional and immunoregulatory phenotype rather than a stable neutrophil subset. Whether targeting this pathway can restore immune homeostasis without compromising antimicrobial defense requires further investigation.

### 3.4 Neutrophil–endothelial communication: DLL4^+^ neutrophils and endothelial Notch1

The DLL4–Notch1 pathway represents an intercellular communication mechanism between neutrophils and pulmonary endothelial cells rather than a Notch1-defined neutrophil phenotype. Delta-like ligand 4 (DLL4) may be induced on activated neutrophils during experimental sepsis, whereas Notch1 functions as the corresponding signaling receptor on pulmonary endothelial cells.

Binding of neutrophil-derived DLL4 to endothelial Notch1 can activate downstream inflammatory signaling and promote endothelial dysfunction, inflammatory cell-death programs, and vascular barrier disruption (Jin et al., 2025). These changes may increase vascular permeability, facilitate further neutrophil recruitment, and amplify pulmonary inflammation. This pathway illustrates how a neutrophil-associated surface phenotype can influence SALI through communication with non-neutrophil cells rather than exclusively through neutrophil-intrinsic mechanisms.

Notably, evidence linking the DLL4–Notch1 axis to endothelial PANoptosis remains emerging and is derived primarily from experimental studies. PANoptosis represents a context-dependent inflammatory cell-death framework involving overlapping features of pyroptosis, apoptosis, and necroptosis. Therefore, this mechanism should be interpreted as a developing hypothesis rather than an established universal pathway in human SALI. Further studies are required to confirm the cellular specificity of DLL4–Notch1 signaling and determine its clinical relevance in sepsis-associated ARDS.

Overall, the reported markers in SALI represent overlapping dimensions of neutrophil biology—including maturation, trafficking, activation, metabolic adaptation, immune regulation, and intercellular communication—rather than a collection of independently validated pathogenic subsets. Their interpretation requires integration of surface phenotype, transcriptional state, anatomical localization, disease stage, and direct functional evidence. This distinction is essential for identifying clinically meaningful neutrophil populations and developing interventions that limit tissue injury while preserving antimicrobial defense.

## 4 Mechanisms and dual role of neutrophils in SALI

Neutrophils represent a central component of the innate immune response and serve as the first line of defense against invading pathogens. In the early stages of sepsis, neutrophils exert protective effects through phagocytosis, degranulation, and the generation of ROS, thereby limiting pathogen dissemination within the lung (Vorobjeva et al., 2021). Maintenance of pulmonary homeostasis depends on the integrity of the alveolar-capillary barrier, which is particularly vulnerable to inflammatory injury during sepsis (D’Agnillo et al., 2021; Zhang et al., 2022). As a result, dysregulated neutrophil responses play a pivotal role in the development of SALI and ARDS, conditions characterized by endothelial dysfunction, increased vascular permeability, and impaired gas exchange (Kempf et al., 2022).

Although neutrophils are essential for host defense, persistent inflammatory stimulation and dysregulated signaling during sepsis drive a shift from protective responses to pathological activation. This transition underlies the dual role of neutrophils in SALI, in which antimicrobial functions become coupled with tissue-damaging and immunoregulatory effects (Liew and Kubes, 2019; Zhu C. L. et al., 2022).

### 4.1 Protective functions in early infection

During the early phase of infection, neutrophils contribute to pathogen clearance through coordinated antimicrobial mechanisms. Phagocytosis and intracellular killing are mediated by both oxygen-dependent and oxygen-independent pathways. Activation of NADPH oxidase promotes the generation of ROS, including superoxide anions and hydrogen peroxide, while antimicrobial peptides and enzymes such as defensins, lysozyme, and myeloperoxidase enhance bactericidal activity (Kosyreva et al., 2024). In addition, interactions between neutrophils and platelets facilitate the formation of platelet-neutrophil complexes, which promote neutrophil recruitment and activation at sites of infection (Mutthineni et al., 2021; Yan et al., 2025).

Neutrophils also form NETs, which consist of chromatin fibers decorated with antimicrobial proteins. These structures trap and immobilize pathogens, particularly those that are difficult to phagocytose, thereby limiting their dissemination and contributing to early immune defense (Huang et al., 2022; Reshetnyak and Nurbaeva, 2023).

### 4.2 Transition to pathological activation

With ongoing sepsis, the neutrophil response gradually shifts from controlled antimicrobial defense to maladaptive activation. This transition is driven by sustained exposure to pathogen-associated molecular patterns (PAMPs) and damage-associated molecular patterns (DAMPs), together with persistent stimulation by inflammatory cytokines and chemokines (Englert et al., 2019; Liew and Kubes, 2019). Under these conditions, the regulatory mechanisms that normally constrain neutrophil activation and promote timely resolution become progressively impaired. As a result, neutrophils exhibit prolonged survival, excessive recruitment, aberrant retention in lung tissue, and altered functional programming (Englert et al., 2019; Liew and Kubes, 2019).

This maladaptive state is characterized not only by enhanced effector responses, such as excessive ROS production, degranulation, and NET release, but also by a loss of spatial and temporal precision in host defense (Santos et al., 2012; Granja et al., 2024). Instead of remaining confined to pathogen clearance, activated neutrophils begin to damage the alveolar epithelium, pulmonary endothelium, and microvascular environment, thereby contributing to barrier dysfunction and edema formation (Leliefeld et al., 2016; Denning et al., 2019). At the same time, persistent inflammatory signaling reshapes neutrophil interactions with macrophages, platelets, endothelial cells, and lymphocytes, further amplifying cytokine release, microthrombosis, and immune dysregulation (Semeraro et al., 2011; Englert et al., 2019).

Importantly, this transition reflects not merely “overactivation,” but a broader functional reprogramming of neutrophils during sepsis. Protective antimicrobial mechanisms become uncoupled from effective resolution, while tissue-damaging and immunosuppressive effects become increasingly prominent (Le-Barillec et al., 1999; Roghanian et al., 2006; Pillay et al., 2012). This shift provides the mechanistic basis for the dual role of neutrophils in SALI and underlies their contribution to lung injury progression.

### 4.3 Mechanisms of neutrophil-mediated lung injury

During SALI, neutrophils exacerbate pathological lung damage by directly inflicting tissue injury and indirectly orchestrating aberrant inflammatory signaling and immune cell interactions. These deleterious effects are executed through the following interconnected mechanisms.

#### 4.3.1 Oxidative and proteolytic tissue injury

Neutrophils release potent ROS and proteolytic enzymes that actively contribute to lung tissue damage, barrier disruption, and immune suppression. Specifically, neutrophil elastase (NE) mediates the degradation of junctional adhesion molecule C (JAM-C) on endothelial cells. This proteolytic cleavage also facilitates aberrant reverse transmigration of neutrophils back into the bloodstream (Colom et al., 2015). Furthermore, ROS generated by a specific subset of neutrophils (characterized by a CD11c^high^/CD62L^low^/CD11b^high^/CD16^high^ phenotype) coupled with Mac-1 integrin activation, significantly elevate hydrogen peroxide levels at the immunological synapse (Pillay et al., 2012). Alongside direct tissue injury, neutrophil-derived serine proteases and NE proteolytically cleave critical immune receptors, such as degrading CD14 on monocytes (Le-Barillec et al., 1999) and inhibiting the expression of costimulatory molecules (CD40, CD80, and CD86) on dendritic cells (Roghanian et al., 2006), thereby fundamentally impairing systemic host immunity.

#### 4.3.2 NET-driven tissue damage and immunothrombosis

The extrusion of NETs is a critical driver of structural and functional pulmonary impairment. Upon activation, NETs can accumulate in the alveolar space, where they promote alveolar–capillary barrier injury, impair alveolar ventilation, and consequently compromise gas exchange (Shi et al., 2021; Corry et al., 2022). Beyond physical blockade, the molecular components of NETs are highly pathogenic; for instance, mitochondrial DNA (mtDNA) embedded within NETs is recognized by alveolar macrophages via the TLR9/STING signaling pathway, thereby promoting the continuous release of pro-inflammatory cytokines and establishing a vicious cycle of inflammation (Zhou et al., 2024; Liang et al., 2025). The pathological burden of NETs is intrinsically linked to cell death pathways, as targeted inhibition of gasdermin D (GSDMD) by disulfiram, or genetic knockout of GSDMD and caspase-11, significantly curtails NET extrusion, protects against cecal ligation and puncture (CLP)-induced sepsis, and improves overall survival (Silva et al., 2021).

#### 4.3.3 Inflammatory amplification and cytokine networks

In sepsis-induced ARDS, pulmonary pathogens initially activate the innate immune response of epithelial cells and alveolar macrophages. This triggers robust neutrophil infiltration and monocyte recruitment, which profoundly accelerate disease progression (Englert et al., 2019; Jacques and Apedaile, 2020). This cellular influx drives the cascade release of diverse pro-inflammatory mediators and chemokines-including IL-1α, IL-1β, IL-6, TNF-α, IL-18, IL-17A, monocyte chemoattractant protein-1 (MCP-1), and high mobility group box 1 (HMGB-1)-culminating in a lethal cytokine storm. The excessive accumulation of these cytokines dismantles alveolar-capillary barrier homeostasis, induces surfactant dysfunction, and causes severe alveolar edema (Englert et al., 2019). Subsequently, the localized pro-inflammatory milieu within the alveolar fluid spills over into the circulatory system, further amplifying the systemic immune response and exacerbating generalized host injury (Mayes-Hopfinger et al., 2021; Wu et al., 2023).

#### 4.3.4 Immune dysregulation and impaired host defense

In severe sepsis, the dysregulation of neutrophil spatiotemporal dynamics and phenotypic alterations severely compromise anti-infective immunity, increasing the risk of secondary pulmonary infections. A hallmark of this dysregulation is impaired directional migration: neutrophils fail to efficiently reach the primary infection site and instead undergo CCR2-mediated aberrant sequestration in distant tissues or organs (Souto et al., 2011). Simultaneously, the upregulation of surface CXCR4 drives pathological accumulation in the lungs (Shen et al., 2021), while other subsets undergo reverse migration back into the systemic circulation (Wang et al., 2017). Concurrently, the septic state stimulates the bone marrow to release a massive influx of immature neutrophils. Compared to their mature counterparts, these immature cells exhibit fundamentally inferior antimicrobial defense capabilities, a defect associated with the downregulation of functional neutrophil genes (Tang et al., 2007; Drifte et al., 2013). Functionally, neutrophils from patients with septic shock highly express arginase 1, which depletes environmental arginine essential for T lymphocyte differentiation, resulting in reduced CD3 expression and suppressed T cell proliferation (Darcy et al., 2014).

#### 4.3.5 Crosstalk with inflammatory cell death pathways

Neutrophil-mediated injury is deeply intertwined with pyroptosis, an inflammatory form of programmed cell death initially characterized in macrophages (Zhu C. L. et al., 2022) but now extensively documented in neutrophils (Mankan et al., 2012; Ryu et al., 2017). During sepsis-induced ARDS, inflammatory caspases (caspase-1, 4, 5, and 11) cleave GSDMD. The liberated N-terminal fragment oligomerizes and perforates the plasma membrane, driving the catastrophic release of bioactive IL-1β, IL-18, and other pro-inflammatory mediators (Magupalli et al., 2021; Mayes-Hopfinger et al., 2021; Christgen et al., 2022). This process is further exacerbated by the gut-lung axis: gut-derived infectious agents and inflammatory mediators can enter the circulation, further increasing intestinal permeability via pyroptosis regulation (Jin et al., 2021; Kerr et al., 2022). These signals subsequently promote canonical and non-canonical inflammasome activation in the lungs, leading to GSDMD-dependent alveolar-capillary barrier disruption and alveolar macrophage pyroptosis (Jin et al., 2021; Jia et al., 2022). Furthermore, the aforementioned accumulation of NETs in the alveolar space acts as a positive feedback loop, directly promoting further caspase-dependent pyroptosis and solidifying the lethal crosstalk between varied cell death modalities (Shi et al., 2021; Corry et al., 2022).

## 5 Natural products regulating neutrophil-related pathological processes in SALI

Natural products (NPs) and their derivatives have attracted increasing attention as potential therapeutic agents for SALI, owing to their anti-inflammatory, antioxidant, and tissue-protective properties (Li et al., 2025). Accumulating evidence indicates that many NPs exert their effects, at least in part, by modulating neutrophil responses, including recruitment, activation, oxidative stress, and NET formation.

Rather than acting through a single pathway, these compounds often regulate multiple aspects of neutrophil function, thereby influencing the balance between antimicrobial defense and tissue injury. This multifaceted regulation is particularly relevant in SALI, where neutrophil dysregulation represents a central pathogenic mechanism.

Based on the eligibility criteria described above, selected natural products associated with the modulation of neutrophil-related mechanisms relevant to SALI are summarized in Table 2.

**TABLE 2:** Natural products associated with modulation of neutrophil-related mechanisms in SALI.

Natural product	Experimental model	Regimen	Neutrophil assessment	Mechanism/target	Main outcome	Clinical evidence	Evidence category	Key limitations	References
TGG	CLP mice	10–40 mg/kg i.p.; daily ×3 before CLP; assessed at 12 h	scRNA-seq/flow cytometry; dHL-60 assays	IFIT1 activation; NF-κB/ROS suppression	Reduced lung inflammation and injury	None	Direct neutrophil-targeting	Pretreatment; acute endpoint; dHL-60 mechanism	Zhao et al. (2026)
BISA	CLP mice	50–200 mg/kg orally; 1 h before CLP	Phagocytosis; peritoneal PMNs; lung MPO	Enhanced phagocytosis; target undefined	Reduced bacterial burden; improved survival	None	Neutrophil-associated	Pretreatment; pulmonary mechanism unclear	Cavalcante et al. (2020)
GE05	LPS-induced ALI mice	250 mg/kg orally; daily ×7 before LPS	Lung MPO; neutrophil-related chemokines	IL-23/IL-17A axis; PI3K/JNK	Reduced inflammation, edema, and mortality	None	Neutrophil-associated	Pretreatment; macrophage-based mechanism	Dong et al. (2025)
Xuebijing injection	CLP mice	5–20 mL/kg i.p.; 2 and 12 h after CLP, then daily	Ly6G/MPO; CitH3; cfDNA; cellular NET assays	CEACAM1/TIM3–ROS–NET axis	Reduced NETs and lung injury; improved function	None	Direct neutrophil-targeting	Undefined components; limited causal validation	Zhang et al. (2025)
eCS	LPS-induced ALI mice; LPS/D-GalN sepsis mice	0.1–1 mg/kg i.t.; 2 h after challenge	BALF neutrophils; lung MPO	Nrf2/NQO1/HO-1/GCLC activation	Reduced lung inflammation; improved survival	None	Neutrophil-associated	Different models; non-neutrophil Nrf2 assays	Han et al. (2019)
Taohe Chengqi decoction	CLP mice	2 or 4 g/kg orally; daily ×7 before CLP	NET/PAD4 assays; PAD4 overexpression	PAD4-mediated NET inhibition	Reduced NETs and lung injury; improved survival	None	Direct neutrophil-targeting	Pretreatment; mainly HL-60 cells; docking evidence	Xie et al. (2025)
SH003	LPS-induced ALI mice	200 or 400 mg/kg orally; 3×/week for 2 weeks before LPS	Human-neutrophil NET assays; lung NET markers	NF-κB/MAPK-related NET inhibition	Reduced NETs, inflammation, and mortality	None	Direct neutrophil-targeting	Pretreatment; LPS model; target undefined	Kim et al. (2024)
Kaurenoic acid	LPS-induced ALI mice; LPS/D-GalN sepsis mice	0.3–30 mg/kg i.t.; 2 h after challenge	BALF neutrophils; lung MPO	Nrf2 activation	Reduced neutrophil infiltration and mortality	None	Neutrophil-associated	Different models; no cell-specific validation	Kim et al. (2016)
Curcumin	CLP rats	100 mg/kg i.p.; 1 h before CLP	Ly6G^+^PD-L1^+^ cells; MPO; neutrophil–T-cell assays	Neutrophil PD-L1 suppression	Reduced lung injury and T-cell apoptosis; improved survival	None	Direct neutrophil-targeting	Pretreatment; high dose; limited causal validation	Zou et al. (2025)
Rosavin	CLP mice	2.5 or 5 mg/kg i.p.; after CLP	BALF neutrophils; MPO/histone staining; NET markers	MAPK-associated NET suppression	Reduced NETs, inflammation, and lung injury	None	Neutrophil-associated	Exact timing unclear; whole-lung signaling	Gao et al. (2023)
Astragaloside IV	CLP mice; LPS-induced sepsis mice	20 mg/kg i.p. or i.v.; daily ×3 before modeling	Neutrophil NET markers; NF-κB; coculture assays	IκBα/NF-κB-dependent NET inhibition	Reduced NET-associated injury; nanoparticles improved survival	None	Direct neutrophil-targeting	Pretreatment; free-drug and nanoparticle effects differ	Wu et al. (2025)
Neutrophil membrane-coated astragaloside IV nanoparticles (As@ZM)	CLP mice	20 mg/kg, i.v., once daily for 3 days before CLP	Neutrophil targeting and NET markers directly assessed	Neutrophil-targeted delivery of astragaloside IV; IκBα/NF-κB inhibition	Greater NET suppression, 7-day survival than free astragaloside IV	None	Direct neutrophil-targeting	Preclinical pretreatment design	Wu et al. (2025)
Dihydromyricetin	CLP mice	100 or 150 mg/kg orally; daily ×3 from 2 h after CLP	BALF neutrophils; lung MPO	NLRP3/caspase-1/GSDMD suppression	Reduced inflammation, edema, and lung injury	None	Neutrophil-associated	Whole-lung mechanism; no direct neutrophil assay	Wang et al. (2019)
Zingerone	CLP mice	25 or 50 mg/kg i.p.; daily from 3 days before to 4 days after CLP	Neutrophil NET/ROS assays; H3Cit; MPO–DNA	Nrf2/ROS-dependent NET inhibition	Reduced NETs, organ injury, and mortality	None	Direct neutrophil-targeting	Includes pretreatment; pharmacological Nrf2 validation	Zhu Y. et al. (2022)
Curdione	CLP mice	50 or 100 mg/kg i.p.; immediately after CLP	Platelet–neutrophil interactions; infiltration; NETs	Platelet-mediated inhibition of recruitment and NETs	Reduced NETs, inflammation, and barrier injury	None	Neutrophil-associated	Primarily platelet-mediated; no survival evidence	Yang K. et al. (2023)
Osthole	LPS-induced ALI mice	20 or 40 mg/kg i.p.; before LPS	Leukocyte recruitment; lung MPO	NF-κB suppression	Reduced inflammation, lung injury, and mortality	None	Indirect	Pretreatment; macrophage/whole-lung mechanism	Jin et al. (2018)
JHQG	LPS-induced ALI mice	3–12 g/kg orally; 2 h after LPS	Ly6G^+^ cells; apoptosis; caspase-3/7; Mcl-1/Bcl-xL	Neutrophil apoptosis; pulmonary TLR4/MyD88/NF-κB suppression	Reduced lung injury; modest survival benefit	None	Neutrophil-associated	High dose; whole-lung signaling; no causal validation	Zhu et al. (2023)

Abbreviations: ALI, acute lung injury; BALF, bronchoalveolar lavage fluid; CLP, cecal ligation and puncture; NET, neutrophil extracellular trap. Evidence categories: Direct neutrophil mechanistic evidence indicates that neutrophils were directly examined and cell-specific pharmacological or mechanistic effects were demonstrated; neutrophil-associated evidence indicates altered neutrophil recruitment, function, apoptosis, or NET-related measures without establishing neutrophils as the exclusive target; indirect evidence indicates that neutrophil involvement was inferred from MPO, activity, inflammatory infiltration, cytokine levels, or histopathological findings. “None” indicates that no clinical evidence was identified for the specific compound in SALI.

### 5.1 1,3,6-Tri-O-Galloyl-β-D-glucose (TGG)

TGG is a polyphenolic gallotannin with reported anti-inflammatory and antioxidant properties. A recent study directly evaluated TGG in experimental sepsis and identified neutrophil IFIT1 as a potential pharmacological target (Zhao et al., 2026). IFIT1 expression was higher in the lung tissues of surviving septic mice, whereas its suppression enhanced NF-κB activation and ROS production in neutrophil-like cells, suggesting a role in limiting excessive neutrophil activation. Consistently, TGG administration reduced pro-inflammatory cytokines in bronchoalveolar lavage fluid and attenuated lung injury in a CLP mouse model in a dose-dependent manner (Zhao et al., 2026). These findings provide direct preclinical evidence linking TGG-mediated IFIT1 regulation to reduced neutrophil hyperactivation and septic lung injury; however, clinical validation remains unavailable.

### 5.2 (-)-α-Bisabolol (BISA)

BISA is an unsaturated monocyclic sesquiterpene predominantly found in the essential oil of chamomile (Moura Rocha et al., 2010). It has attracted increasing attention due to its anti-inflammatory and antioxidant properties, with emerging evidence suggesting a role in modulating neutrophil function in SALI. BISA has been reported to regulate inflammatory responses and improve survival in experimental sepsis models. In CLP-induced systemic infection, BISA treatment reduces myeloperoxidase activity in lung tissue, indicating decreased neutrophil infiltration and activation. This is accompanied by reduced production of pro-inflammatory mediators, including TNF-α, IL-1β, IL-6, macrophage inflammatory protein-2, and nitric oxide (Cavalcante et al., 2020). In addition, BISA enhances neutrophil phagocytic activity and modulates leukocyte chemotaxis, suggesting a role in restoring effective host defense while limiting excessive inflammation. Further studies are required to evaluate its pharmacological properties and potential applicability in clinical settings.

### 5.3 Gastrodia elata extract (GE05)

GE05 is extracted from the rhizome of gastrodia elata and contains several major constituents, including gastrodin, gastrodin B, parishin, and hydroxybenzyl alcohol (Dong et al., 2025). Although gastrodia elata has traditionally been used for neurological disorders such as headache and neuralgia (Zhan et al., 2016), increasing evidence suggests that it also possesses antibacterial, antiviral, and anti-inflammatory activities (Dong et al., 2025).

Its protective effects in SALI appear to be closely related to suppression of the IL-23/IL-17A axis, a pathway that contributes to neutrophil recruitment and inflammatory amplification. IL-17A promotes the production of chemokines and colony-stimulating factors, thereby enhancing neutrophil infiltration and activating downstream inflammatory pathways such as PI3K and JNK (Miossec and Kolls, 2012; Pinart et al., 2013; Gaffen et al., 2014). In experimental models, GE05 reduces neutrophil accumulation in lung tissue, inhibits PI3K/JNK signaling, and lowers the production of pro-inflammatory cytokines, ultimately attenuating lung injury (Dong et al., 2025).

### 5.4 Xuebijing injection

Xuebijing Injection is a standardized traditional chinese medicine formulation with a clinical and regulatory background distinct from that of the predominantly experimental natural products discussed above. It has been approved in China as an adjunctive treatment for sepsis and has been evaluated in clinical studies primarily for general sepsis-related outcomes, including organ dysfunction, disease severity, and mortality (Huang et al., 2011; Liu S. et al., 2023). However, evidence for its effects on pulmonary or ARDS-specific outcomes is comparatively limited, and the extent to which any clinical benefit is mediated specifically through neutrophils or NET formation remains uncertain.

Experimental studies suggest that Xuebijing may modulate neutrophil-associated oxidative stress and NET formation. The CEACAM1/TIM3 pathway has been implicated in the regulation of neutrophil activation, while CEACAM1-related signaling may interact with the S1PR2/S1PR3 axis and influence NET-associated responses (Ye et al., 2018; Hirao et al., 2023). In an experimental model of SALI, Xuebijing administration was associated with altered CEACAM1/TIM3 signaling, reduced ROS production, decreased NET-associated markers, and alleviation of alveolar–capillary barrier injury (Zhang et al., 2025). These findings suggest a potential association between Xuebijing, CEACAM1/TIM3-related signaling, and pathological neutrophil responses. Nevertheless, unless pathway-specific blockade, rescue, or neutrophil-specific experiments establish causality, the CEACAM1/TIM3 pathway should be considered a candidate mechanism rather than a confirmed mediator of the clinical effects of Xuebijing. Therefore, current evidence supports a distinction between its reported clinical effects in sepsis and its proposed neutrophil- and NET-associated mechanisms derived mainly from experimental models.

### 5.5 50% ethanol extract of Chung-Sang (eCS)

Chung-Sang (CS) is a multi-herb decoction traditionally utilized to alleviate inflammatory respiratory conditions (Han et al., 2019). Its ethanol extract (eCS) has demonstrated potent anti-inflammatory effects in the lung, primarily through the activation of the nuclear factor erythroid 2-related factor 2 (Nrf2) antioxidant pathway. In LPS-induced acute lung injury models, eCS treatment profoundly upregulates Nrf2-dependent cytoprotective genes (such as HO-1, NQO-1, and GCLC). Concurrently, this antioxidant enhancement is coupled with a marked reduction in pulmonary myeloperoxidase (MPO) activity, indicating a significant decrease in neutrophil infiltration and sequestration within the lung tissue. These findings suggest that eCS mitigates neutrophil-dominated pulmonary inflammation by enhancing the host’s endogenous antioxidant defense mechanisms, presenting a viable strategy for SALI intervention (Han et al., 2019).

### 5.6 Taohe Chengqi decoction

Taohe Chengqi Decoction, a classical formula from the treatise on febrile diseases, is traditionally employed to promote blood circulation and resolve stasis (Lu et al., 2024; Yuan et al., 2024). Beyond its systemic microcirculatory benefits, this decoction exerts targeted regulatory effects on neutrophil recruitment and effector functions during sepsis. A critical mechanism underlying its efficacy in SALI is the suppression of the CXCL2/CXCR2 chemokine axis, which serves as a primary driver for neutrophil and monocyte chemotaxis to inflammatory sites. By downregulating CXCR2 and its ligand CXCL2, the decoction significantly limits abnormal neutrophil infiltration into the lung interstitium (Xie et al., 2025). Furthermore, Taohe Chengqi decoction actively inhibits peptidylarginine deiminase 4 (PAD4)-mediated NETosis, reducing the extracellular release of citrullinated histone H3, NE, and MPO. Notably, glycyrrhizin, a key active component of the decoction, also contributes to lung protection by blocking the HMGB1/TLR9 pathway, further restraining NET formation (Gu et al., 2022; Xie et al., 2025). Together, these multifaceted mechanisms highlight its potential to interrupt the vicious cycle of neutrophil recruitment and tissue destruction.

### 5.7 SH003 (novel TCM compound)

SH003 is an optimized traditional chinese medicine formulation comprising immunomodulatory herbs such as Astragalus membranaceus, Angelica sinensis, and Trichosanthes kirilowii (Lee et al., 2021; Lu et al., 2022). It has been demonstrated to correct neutrophil imbalance by reversing phenotypic alterations induced by sepsis, specifically mitigating the pathological upregulation of CXCR4 and the downregulation of CXCR2, thereby restoring normal neutrophil mobilization (Eash et al., 2009; Kim et al., 2024). Beyond modulating migration, SH003 functions as a potent cytokine storm inhibitor, significantly reducing plasma levels of IL-1β, IL-6, TNF-α, and key chemokines (MCP-1, CXCL1/2). At the effector level, it directly suppresses NET formation by downregulating the expression of PAD4 and NE in lung tissue. By concurrently regulating neutrophil migration, cytokine release, and NET generation, SH003 provides a comprehensive pharmacological approach for mitigating sepsis-induced lung injury (Kim et al., 2024).

### 5.8 Kaurenoic acid (KA)

Kaurenoic acid (ent-kaur-16-en-19-oic acid) is a bioactive diterpene isolated from the roots of Aralia continentalis (Lim et al., 2009). It is characterized by its remarkable potency, capable of exerting profound anti-inflammatory effects even at nanomolar concentrations. The therapeutic mechanism of KA is highly dependent on the activation of the Nrf2 transcription factor (Lyu et al., 2011). In experimental SALI, targeted intrapulmonary or tracheal administration of KA efficiently halts neutrophil migration into the alveolar space and downregulates pro-inflammatory gene expression, ultimately improving survival rates. Crucially, these protective effects are completely abrogated in Nrf2-knockout models, confirming that KA prevents neutrophilic lung destruction specifically by driving Nrf2-mediated antioxidant and anti-inflammatory pathways (Kim et al., 2016).

### 5.9 Curcumin

Curcumin is a pleiotropic phytochemical derived from curcuma species, widely recognized for its antioxidant and anti-inflammatory properties (He et al., 2015; Shafabakhsh et al., 2019). In the context of sepsis, an emerging pathological feature is the upregulation of the immune checkpoint molecule PD-L1 on neutrophils. This upregulation delays neutrophil apoptosis while concurrently inducing T-cell exhaustion, thereby promoting an immunosuppressive state (Wang J. F. et al., 2021; Li et al., 2024; Liu et al., 2024; Arapis et al., 2025). Curcumin directly addresses this dysregulation. Experimental studies show that curcumin administration during sepsis significantly reduces PD-L1 expression on neutrophils in both circulation and lung tissue. This downregulation not only shortens the lifespan of hyperactivated neutrophils but also prevents PD-L1-mediated apoptosis of T lymphocytes. By reversing this immune checkpoint abnormality, curcumin restores systemic inflammatory balance (decreasing TNF-α/IL-6 while increasing IL-10) and prevents secondary immunosuppression in SALI (Zou et al., 2025).

### 5.10 Rosavin

Rosavin, an alkyl phenyl diglycoside isolated from rhodiola rosea, exhibits significant protective effects against pulmonary inflammation (Panossian et al., 2010). Its protective mechanism in SALI is primarily mediated through the inhibition of the MAPK signaling cascade, including ERK, p38, and JNK. During sepsis, hyperactivation of the MAPK pathway promotes intense effector functions in neutrophils. Administration of rosavin markedly reduces the phosphorylation levels of these kinases, thereby attenuating neutrophil hyperactivation and significantly decreasing NET formation in the bronchoalveolar space (Gao et al., 2023). By inhibiting this signaling axis, rosavin mitigates the cytotoxic effects of NET components on pulmonary epithelial cells, thereby preserving lung function.

### 5.11 Astragaloside IV

Astragaloside IV, a bioactive saponin isolated from astragalus membranaceus, exhibits anti-inflammatory and immunomodulatory properties. In experimental LPS- and CLP-induced sepsis models, astragaloside IV reduced pulmonary inflammatory-cell infiltration and pro-inflammatory cytokine expression and alleviated histological lung injury. These anti-inflammatory effects have been associated with suppression of NF-κB and AP-1 activation (Zhang and Frei, 2015; Yang H. et al., 2023). More recently, astragaloside IV was shown to interact with IκBα and inhibit its phosphorylation, thereby reducing NF-κB p65 activation and NET formation in neutrophils. Treatment decreased NET-associated markers, including CitH3–DNA and MPO–DNA complexes, while conditioned medium from astragaloside IV-treated neutrophils reduced oxidative stress and cell death in pulmonary endothelial and epithelial cells. These findings suggest that its protective effects in sepsis may partly involve attenuation of NF-κB-dependent NET formation and subsequent NET-associated pulmonary injury. Notably, a neutrophil membrane-coated nanoparticle formulation of astragaloside IV further enhanced drug stability, bioavailability, NET suppression, organ protection, and survival in septic mice. Nevertheless, these findings are derived from experimental models, and the clinical efficacy and pulmonary specificity of this mechanism remain to be established (Wu et al., 2025).

### 5.12 Dihydromyricetin (DHM)

Dihydromyricetin is a naturally occurring flavonoid that has garnered attention for its ability to modulate oxidative stress and programmed cell death (Wu et al., 2022). In SALI, activation of the NLRP3 inflammasome within immune cells exacerbates inflammatory amplification and apoptosis. DHM counteracts this process by stimulating the Nrf2 signaling pathway, which provides critical antioxidant defense and subsequently suppresses NLRP3 inflammasome activation (Xu et al., 2024). Through this mechanism, DHM reduces pathological neutrophil apoptosis and systemic inflammatory responses, thereby effectively protecting the pulmonary microenvironment from ROS and inflammasome-driven tissue damage (Wang et al., 2019).

### 5.13 Zingerone (ZIN)

Zingerone is an active component found in ginger (Zingiber officinale) with potent anti-inflammatory and antioxidant properties (Ali et al., 2008; Ahmad et al., 2015). Given that ROS generation is a prerequisite for most forms of NET formation, Zingerone’s strong antioxidant capacity positions it as a compelling candidate for selective antioxidant therapy in SALI (Liu et al., 2019; Zhu et al., 2021). Pretreatment with zingerone significantly suppresses stimulus-induced ROS bursts in neutrophils and the subsequent NET release. Mechanistically, this inhibitory effect is entirely dependent on the activation of Nrf2; pharmacological blockade of Nrf2 abrogates zingerone’s ability to inhibit ROS and NETs. Consequently, zingerone reduces inflammatory cell infiltration, hemorrhage, and interstitial edema in septic lungs by disrupting the ROS-NET pathological axis (Zhu Y. et al., 2022).

### 5.14 Curdione

Curdione, derived from curcuma phaeocaulis, is characterized by its synergistic anti-inflammatory and antiplatelet aggregation properties (Yang K. et al., 2023). In SALI, the dynamic interaction between activated platelets and neutrophils is a key driver of pulmonary infiltration and NETosis. Curdione targets this early event by inhibiting platelet activation (potentially via AMPK-integrin pathways) (Fang et al., 2019), thereby interrupting platelet-neutrophil interactions. This upstream blockade effectively prevents the subsequent recruitment, sequestration, and NET generation of neutrophils within the lung tissue. The resulting attenuation of pulmonary oxidative stress and cytokine storm highlights curdione as a promising agent for managing platelet-neutrophil-driven lung injury (Yang K. et al., 2023).

### 5.15 Osthole

Osthole, a natural coumarin derivative, exhibits a broad spectrum of pharmacological actions, primarily attributed to its modulation of cyclic nucleotides (cAMP/cGMP) and downstream inflammatory cascades (Zhang et al., 2015; Shokoohinia et al., 2018). In septic models, osthole acts as a potent inhibitor of the NF-κB signaling pathway. Administration of osthole effectively suppresses the secretion of major pro-inflammatory cytokines (IL-6, TNF-α) and markedly reduces MPO activity in lung tissue. By diminishing neutrophil recruitment to the lungs and their subsequent inflammatory responses, osthole ameliorates alveolar-capillary damage, demonstrating significant therapeutic potential for SALI (Jin et al., 2018).

### 5.16 Jinhua Qinggan granules (JHQG)

Jinhua Qinggan Granules, a patented TCM initially developed for viral respiratory infections, also demonstrate profound therapeutic efficacy in bacterial and endotoxin-induced lung injury (Wang C. et al., 2021; Yao et al., 2023). During sepsis, delayed neutrophil apoptosis prolongs the inflammatory phase and exacerbates tissue damage. JHQG functions by blocking the TLR4/MyD88/NF-κB signaling axis. Notably, while inhibiting the production of pro-inflammatory cytokines (TNF-α, IL-1β, IL-6), this blockade also promotes the timely apoptosis of hyperactivated neutrophils. By facilitating the physiological clearance of neutrophils, this preparation contributes to inflammation resolution and improves survival in septic models, offering a host-directed strategy for the management of SALI (Zhu et al., 2023).

## 6 Discussion

SALI remains a devastating complication of sepsis with high morbidity and mortality, while clinically effective targeted therapies are still limited (Vincent et al., 2014; van Vught et al., 2016). In this context, neutrophils have emerged as central regulators of SALI pathogenesis because they not only constitute the first line of antimicrobial defense but also represent major drivers of inflammatory amplification, tissue destruction, immunothrombosis, and immune dysregulation (Vorobjeva et al., 2021; Xu et al., 2025). The evidence summarized in this review indicates that neutrophils in SALI should no longer be regarded as a homogeneous population. Instead, they exhibit dynamic and functionally diverse states shaped by maturation, anatomical localization, disease stage, and the pulmonary microenvironment. Chemokine receptors, adhesion molecules, immune checkpoint proteins, and metabolic mediators collectively regulate neutrophil trafficking, activation, survival, NET formation, and communication with endothelial cells, epithelial cells, platelets, and lymphocytes. Importantly, these molecular features may represent overlapping and context-dependent phenotypes rather than discrete, stable neutrophil subsets.

This framework helps reconcile the apparently opposing roles of neutrophils during sepsis. Early after infection, phagocytosis, oxidative burst, degranulation, and appropriately controlled NET formation contribute to pathogen containment (Vorobjeva et al., 2021). With persistent or excessive septic stimulation, however, these protective responses may become maladaptive (Liew and Kubes, 2019; Zhu C. L. et al., 2022). Excessive neutrophil recruitment, prolonged survival, aberrant retention in the lung, uncontrolled ROS release, protease-mediated barrier injury, exaggerated NETosis, and crosstalk with pyroptotic pathways together contribute to alveolar–capillary barrier failure, pulmonary edema, microvascular thrombosis, and defective resolution of inflammation (Colom et al., 2015; Shi et al., 2021; Corry et al., 2022; Kempf et al., 2022; Zhu C. L. et al., 2022). Importantly, this process reflects not merely overactivation, but a broader functional reprogramming of neutrophils during sepsis (Englert et al., 2019; Liew and Kubes, 2019).

The markers discussed in this review illustrate different, partially overlapping aspects of this reprogramming (Janicova and Relja, 2021). CXCR4, CXCR2, and CD11b primarily reflect maturation, trafficking, adhesion, or activation; CD16 and CD177 identify functional surface phenotypes whose significance depends on the accompanying marker profile and biological context; Fth1^hi^, Prok2^hi^, CD64^+^CD271^+^, and PD-L1^+^ cells represent disease-associated transcriptional or functional states; and the DLL4–Notch1 axis exemplifies neutrophil–endothelial communication rather than a Notch1-defined neutrophil subset. This distinction is important for therapeutic interpretation. Future interventions should be guided by the location, timing, and function of the targeted neutrophil state instead of relying on a single marker or broadly suppressing the entire neutrophil compartment.

The phase-dependent role of neutrophils also creates a narrow therapeutic window. Inhibiting recruitment, oxidative burst, NET formation, or survival may reduce tissue injury when these processes are excessive, but broad or premature suppression may compromise bacterial clearance and increase susceptibility to secondary infection, particularly when pathogen burden remains high or source control is incomplete. Therapeutic effects are therefore likely to depend on disease stage, immune phenotype, anatomical compartment, timing of administration, and whether an intervention is used prophylactically or after lung injury has developed. The clinically relevant goal is not generalized neutrophil inhibition, but timely restoration of balanced antimicrobial and inflammatory functions.

Within this context, natural products and traditional herbal formulations represent a potential source of multitarget therapeutic candidates. The agents reviewed here have been reported to modulate neutrophil-related processes, including chemotaxis, adhesion, oxidative stress, inflammatory signaling, apoptosis, platelet–neutrophil interactions, and NET formation. Their reported effects converge on several pathogenic hubs, including CXCR4/CXCR2-dependent trafficking, NF-κB and MAPK signaling, ROS production, PAD4-associated NET formation, and PD-L1-related immune dysregulation (Eash et al., 2009; Wang et al., 2019; Gao et al., 2023; Kim et al., 2024; Wu et al., 2025; Zou et al., 2025). Such multitarget activity may be relevant to the complex pathophysiology of SALI; however, it also complicates identification of the active constituent, direct cellular target, dose–response relationship, and mechanism responsible for protection.

The potential clinical value of these agents must be considered against current evidence-based sepsis management. Corticosteroids have an established adjunctive role in selected patients with septic shock and an ongoing vasopressor requirement (Annane et al., 2018; Venkatesh et al., 2018; Evans et al., 2021), whereas cytokine-targeted strategies directed against TNF-α, IL-1, or IL-6 have not produced consistent benefits in heterogeneous sepsis populations (Fisher et al., 1994; Opal et al., 1997; Abraham et al., 2001). At present, natural products cannot be considered alternatives to established supportive care, timely antimicrobial therapy, source control, lung-protective ventilation, or other guideline-based interventions. Their translation will require standardized preparations, rigorous pharmacokinetic and pharmacodynamic characterization, clinically achievable dosing, systematic assessment of toxicity and drug interactions, and prospective studies using treatment rather than predominantly pretreatment designs.

Future research should integrate single-cell, spatial, and functional approaches to distinguish transient neutrophil states from reproducible disease-associated populations and to define their temporal and anatomical relationships in SALI. Cross-species validation is particularly important because findings from LPS-induced ALI or other reductionist models may not reproduce the immune complexity of polymicrobial sepsis or clinically defined sepsis-associated ARDS. Studies should also incorporate direct measures of neutrophil function, neutrophil-specific perturbation, and clinically relevant endpoints to establish causality. Ultimately, biomarker-guided stratification may help identify patients in whom a particular neutrophil program is dominant and determine when a targeted or combination intervention is most likely to restore immune balance without weakening host defense.

In conclusion, neutrophil dysregulation represents a major pathogenic axis linking inflammation, barrier injury, immunothrombosis, and immune dysfunction in SALI. Viewing neutrophils as dynamic, spatially organized, and functionally diverse states provides a more precise framework than classifying them as fixed subsets on the basis of individual markers. Natural products offer mechanistically interesting candidates for modulating these processes, but their therapeutic promise remains largely preclinical. Progress toward clinical application will depend on stronger causal evidence, model-appropriate validation, standardized pharmacology, and stage-specific therapeutic strategies that preserve essential antimicrobial functions.

## 7 Limitations

Several limitations of this narrative review and the underlying evidence should be considered. First, the literature summarized here spans clinically defined sepsis-associated ARDS, human sepsis without uniform pulmonary phenotyping, polymicrobial sepsis models, LPS-induced ALI, and other inflammatory lung-injury models. Although evidence from non-SALI models can provide mechanistic support, it should not be interpreted as direct confirmation of the same mechanism in polymicrobial sepsis or human sepsis-associated ARDS. The heterogeneity of species, injury models, treatment timing, outcome measures, and terminology also limits direct comparison across studies. In addition, because this was a narrative rather than systematic review, study selection was not exhaustive and the available evidence was not subjected to formal risk-of-bias assessment or quantitative synthesis.

Second, neutrophil heterogeneity remains incompletely defined. Many reported phenotypes are based on single markers, small datasets, or transcriptional clusters that may represent transient activation, maturation, aging, or compartment-specific states rather than stable subsets. Differences in sampling time, tissue compartment, analytical platform, and gating or clustering strategy further limit reproducibility. Most disease-associated states have not been validated across independent cohorts, at the protein level, or by lineage-tracing and functional experiments, and their relevance to human SALI remains uncertain.

Third, the strength and cellular specificity of evidence for the reviewed natural products vary considerably. Some studies directly measured neutrophil migration, phagocytosis, ROS production, apoptosis, or NET formation, whereas others inferred neutrophil involvement from pulmonary MPO activity, histological infiltration, or changes in broadly expressed inflammatory pathways. Several agents may act indirectly through platelets, macrophages, endothelial cells, epithelial cells, or systemic inflammatory responses; thus, neutrophils cannot yet be regarded as their exclusive or direct targets. Most studies are preclinical and frequently use pretreatment designs, while information on active constituents, formulation and batch consistency, bioavailability, pharmacokinetics, clinically relevant dosing, long-term safety, and drug interactions remains limited. Future work using primary neutrophils, neutrophil-specific genetic manipulation, depletion or adoptive-transfer strategies, standardized preparations, and well-designed clinical studies is needed to establish causal mechanisms and translational feasibility.
